# MAPPING THE INTERPLAY OF SOCIAL HEALTH AND COGNITIVE FUNCTIONING: A MIXED RESEARCH KNOWLEDGE SYNTHESIS

**DOI:** 10.1093/geroni/igac059.1708

**Published:** 2022-12-20

**Authors:** Henrik Wiegelmann, Imke Seifert, Marta Lenart-Bugla, Mateusz Łuc, Ansgar Gerhardus, Dorota Szcześniak, Joanna Rymaszewska, Karin Wolf-Ostermann

**Affiliations:** University of Bremen, Bremen, Bremen, Germany; University of Bremen, Bremen, Bremen, Germany; Wroclaw Medical University, Wroclaw, Dolnoslaskie, Poland; Wroclaw Medical University, Wroclaw, Dolnoslaskie, Poland; University of Bremen, Bremen, Bremen, Germany; Wroclaw Medical University, Wroclaw, Dolnoslaskie, Poland; Wroclaw Medical University, Wroclaw, Dolnoslaskie, Poland; University of Bremen, Bremen, Bremen, Germany

## Abstract

**Introduction:**

Dementia is a syndrome with complex underlying bio-psycho-social mechanisms relevant for prevention and intervention. This work presents a mixed research knowledge synthesis, mapping the multidimensional interplay of social health and cognitive functioning in dementia.

**Methods:**

Data integration from 1) systematic review, 2) group model building workshops, (3) iterative integration of multi-national cohort studies (4) ongoing revisions of the social health concept via expert discussions.

**Results:**

The map comprises more than 50 markers, clustered in six domains (social health, psychological pathways, physiological pathways, health behavior pathways, brain/cognitive reserve, cognitive functioning). The social health domain is structured in six sub-domains representing a novel conceptual understanding. Three pathways (physiological, psychological, health behavior) reflect principal mechanisms connecting social health with brain/cognitive reserve and cognitive functioning.

**Conclusion:**

The map depicts dynamic relationships between social health and cognitive functioning that can serve as a basis for recommendations, both for prevention and for improved dementia care.

